# Audiovisual Lexical Retrieval Deficits Following Left Hemisphere Stroke

**DOI:** 10.3390/brainsci8120206

**Published:** 2018-11-28

**Authors:** Brenda Hanna-Pladdy, Hyun Choi, Brian Herman, Spenser Haffey

**Affiliations:** 1 Department of Diagnostic Radiology & Nuclear Medicine, University of Maryland School of Medicine, Baltimore, MD 21201, USA; 2 Department of Clinical Research for Rehabilitation, Korea National Rehabilitation Research Institute, Seoul 01022, Korea; choi1h@korea.kr; 3 Kansas City University of Medicine & Biosciences, Kansas City, MO 64106, USA; brian.herman11@gmail.com; 4 Department of Psychology, University of Maryland Baltimore County (Shady Grove), Rockville, MD 20850, USA; spenserhaffey@gmail.com

**Keywords:** auditory naming, environmental sounds, lexical retrieval, audiovisual, multisensory, left hemisphere stroke

## Abstract

Binding sensory features of multiple modalities of what we hear and see allows formation of a coherent percept to access semantics. Previous work on object naming has focused on visual confrontation naming with limited research in nonverbal auditory or multisensory processing. To investigate neural substrates and sensory effects of lexical retrieval, we evaluated healthy adults (*n* = 118) and left hemisphere stroke patients (LHD, *n* = 42) in naming manipulable objects across auditory (sound), visual (picture), and multisensory (audiovisual) conditions. LHD patients were divided into cortical, cortical–subcortical, or subcortical lesions (CO, CO–SC, SC), and specific lesion location investigated in a predictive model. Subjects produced lower accuracy in auditory naming relative to other conditions. Controls demonstrated greater naming accuracy and faster reaction times across all conditions compared to LHD patients. Naming across conditions was most severely impaired in CO patients. Both auditory and visual naming accuracy were impacted by temporal lobe involvement, although auditory naming was sensitive to lesions extending subcortically. Only controls demonstrated significant improvement over visual naming with the addition of auditory cues (i.e., multisensory condition). Results support overlapping neural networks for visual and auditory modalities related to semantic integration in lexical retrieval and temporal lobe involvement, while multisensory integration was impacted by both occipital and temporal lobe lesion involvement. The findings support modality specificity in naming and suggest that auditory naming is mediated by a distributed cortical–subcortical network overlapping with networks mediating spatiotemporal aspects of skilled movements producing sound.

## 1. Introduction

The formation of a coherent percept requires binding sensory features related to our perception of what we hear (auditory) and see (visual) with semantics. This is distinguished from multisensory integration based on a cognitive decision to integrate auditory–visual information [1]. Integration of sensory information can improve performance, response speed and localization [2]. Although there are few investigations focused on auditory–visual integration during object recognition and naming, there is evidence multisensory presentation may facilitate lexical retrieval in aging and speech recognition [3,4].

Visual object tasks have predominated investigations of neural mechanisms mediating naming and have implicated the left posterior and temporoparietal regions as critical for word retrieval [5,6,7]. Conversely, auditory naming localization has not been well investigated with limited understanding of neural processing pathways for nonverbal sound recognition. Auditory description naming has been associated with disruption to the anterior temporal lobe in epilepsy patients, while both auditory and visual naming have demonstrated impairment following posterior temporal lobe direct cortical stimulation and postsurgical resection [8,9,10].

Language perception involves sensory systems including auditory and visual modalities, but previous studies in language systems have focused on semantics with minimal consideration of sensory systems [11]. The neural circuits underlying language functions involve the inferior frontal cortex (including Broca’s area), the superior temporal cortex (including Wernicke’s area), and the interplay between these regions. Viewing and naming pictures of objects and tools associated with skilled movements (as opposed to other object categories) may selectively activate left ventral premotor and posterior parietal cortex reflecting ventral (“what”) and dorsal (“where”) visual processing streams [12]. Despite evidence suggesting specialized auditory networks processing “what” and “where”, the functional organization of sound recognition has been difficult to characterize in terms of distinct networks [13]. Sound object recognition requires several steps in segregating spatial, temporal, and synchronous cues, and also in semantic identification of the sounds associated with actual objects. 

Lexical retrieval deficits are common and persistent following left hemisphere stroke, but the anatomical factors predicting recovery and compensatory mechanisms remain unclear. In addition to age-related changes in the efficiency of lexical retrieval, naming is frequently impaired in aphasia following a vascular event to the left hemisphere that disrupts cortical–subcortical networks [14]. Voxel-based lesion symptom studies have predicted lexical retrieval deficits based on damage to a cortical–subcortical network involving the left mid-posterior inferior temporal cortex and underlying connections, especially the longitudinal fasciculus [15].

Previous studies have typically relied on visual confrontation naming (i.e., picture-naming) to assess lexical retrieval failures. It is possible that there are differences in visual and auditory naming as well as multisensory integration based on lesion distribution. There is evidence that in addition to damage to the left cortex, the underlying connections contribute to persistent naming deficits, supporting both cortical and subcortical contributions to lexical retrieval [15]. In healthy older adults, auditory naming proved more sensitive to detecting naming failures, but the differences in cortical–subcortical contributions to visual and auditory naming have not been previously investigated [3]. Therefore, it remains unclear how nonverbal processing is impacted post-stroke and whether select patients may benefit from multisensory cues similar to what has been demonstrated in normal aging. 

Findings of numerous studies argue for modality specificity, although the critical regions mediating audiovisual integration in naming are not well established. Conversely, there are numerous investigations focused on audiovisual integration in speech processing that have revealed critical contributions from the auditory cortex, the posterior superior temporal sulcus (STS), and the posterior inferior frontal gyrus [16,17,18]. In addition to these regions, lesion studies have also implicated lateral occipital visual cortex in addition to multisensory regions in the STS as critical to multisensory speech integration [4]. Unlike multisensory speech integration, the overlapping network mediating auditory verbal and nonverbal (sound) naming has not been well-characterized. Thus, the brain regions impacting audiovisual integration in lexical retrieval warrant further elucidation to better characterize lexical retrieval and develop potential intervention models.

Furthermore, it remains unclear how the auditory network overlaps with visual processing networks related to biological motion and visual recognition of tools associated with multisensory processing of object-associated action knowledge [19]. Determination of the more effective unisensory response (e.g., to visual or auditory) in comparison to simultaneous presentation of two sensory stimuli (audiovisual), allows for estimation of gains attributable to multisensory interactions (visual–auditory presentation). It is conceivable that integrating information across modalities may improve behavioral outcomes and response speed [2]. Study findings will help determine whether multisensory integration is a useful approach to facilitate naming in impaired populations such as those with stroke and aphasia. 

The objective of this study was to extend our previous work in auditory and audiovisual naming in aging to left hemisphere stroke. We evaluated how lesions in the dominant hemisphere for language (left hemisphere stroke) impact naming to visual, auditory, and multisensory cues to determine which patients benefit from sensory cues and multisensory information to facilitate lexical retrieval. Related to degree of language deficits following stroke and lesion location, we predicted there would be significant variability across naming conditions elucidating neural mechanisms mediating modality specificity and multisensory integration. These findings may be capable of assisting prediction of which patients will display persisting language deficits following stroke, and allow realistic goals for rehabilitation efforts to reduce disability. 

## 2. Materials and Methods

The protocol conformed to the Declaration of Helsinki and was approved by the Institutional Review Boards (IRB) for the University of Kansas Medical Center (KUMC), Emory University School of Medicine, and the University of Maryland School of Medicine (UMB HP-00060452). All subjects gave written informed consent prior to entering the study, and caregivers were consulted for all stroke patients regarding the details of the experiment prior to their enrollment. 

### 2.1. Participants 

One hundred and sixty older participants comprised of 42 left hemisphere ischemic stroke patients (LHD; 27 males and 15 females) and 118 healthy controls (53 males and 65 females) were recruited from both KUMC and Emory medical centers, and data was processed at UMB. Left hemisphere stroke patients were characterized based on involvement of cortical (CO, *n* = 17), cortical–subcortical (CO–SC, *n* = 10), and subcortical lesion locations (SC, *n* = 15) derived from clinical neuroimaging results (see Table 1 for detailed lesion location based on clinical MRI or CT). Neuroimaging was not obtained on healthy controls. The average age of healthy controls (63.1 years) was slightly higher than stroke patients (60.4 years; see Table 2), but was not statistically significant. Age between lesion groups (SC = 60.9; CO = 63; CO–SC = 57.1 years of age) revealed lower age for CO–SC relative to CO patients. The average educational level of the healthy participants (16.5 years) was higher than stroke patients (13.4 years), but education was not different between lesion groups (SC = 13.1; CO = 13.4; CO–SC = 13.8 years). Age and education were both utilized in the statistical analyses as covariates. 

All subjects were native English speakers, and healthy controls were free of dementia and language impairments (Table 1). Subjects were screened for cognitive deficits with the Mini-Mental Status Examination [21], and also received a comprehensive neuropsychological examination to ensure that healthy controls were free of significant language and cognitive deficits. Handedness was determined by the Edinburgh Handedness Inventory [22], and most participants were right hand dominant. However, five controls and 8 stroke patients were left hand dominant, but the results did not differ between right and left-handed subjects. General exclusion criteria for normal controls and stroke included: (1) chronic, serious medical conditions, (2) other neurological disease, (3) psychiatric disease including current depression and anxiety untreated, or (4) significant substance abuse/dependence. Stroke exclusion criteria included: (1) evidence of bilateral or right hemisphere stroke, or (2) significant confluent white matter abnormalities. Left hemisphere stroke patients were characterized based on involvement of cortical (CO, *n* = 17), cortical–subcortical (CO–SC, *n* = 10), and subcortical lesion locations (SC, *n* = 15) derived from clinical neuroimaging results (see Table 2 for detailed lesion location based on clinical MRI or CT). Lesion stroke interval was calculated based on the number of months that elapsed between the date of the stroke to language examination. The stroke-testing interval range spanned from 4.5 months to 6 years for the majority of the LHD patients, with two patients with intervals extending outside this range (see Table 2 for characteristics of patients enrolled). Stroke interval was utilized as a predictor in the regression models. 

### 2.2. Language, Neuropsychological, and Audiological Assessments

*Hearing and Audiology Screening.* Subjects were screened with the Hearing Handicap Inventory for the Elderly (HHIE-S) [23,24], and an audiometer screening to identify hearing loss that may interfere with processing of auditory stimuli. The hearing screening test was conducted with an audiometer (MA27) at 25 dB at 250, 500, 1000, 2000, 4000, and 8000 Hz bilaterally. This was followed by a threshold test conducted for each ear using the same frequency levels. Eighteen of the 160 enrolled subjects did not receive an audiology evaluation (nine normal controls and nine stroke patients), although the majority of these participants did alternatively complete the HHIE-S (11 of 18) and most (seven of the 11) and responses were consistent with no hearing handicap. Participants were evaluated based on differences between the ears with a goal of obtaining less than 35 dB difference at any frequency level set similar to criteria in previous studies [3,25]. All enrolled controls with the exception of three subjects met this criterion, and since they exceeded at only one frequency and obtained adequate scores on auditory naming, they were not excluded from the study. Audiological assessment was considered in a between groups fashion in the analysis. 

*Language Assessment.* Participants were initially screened for the presence of anomia with the Boston Naming Test and verbal and semantic fluency [26,27]. Language was also characterized by the first part of the Western Aphasia Battery [20] to derive an aphasia quotient (see WAB-AQ Table 1 and Table 2). Of the 42 LHD stroke patients included in the study, 24 stroke patients were classified into one of the aphasia subtypes based on WAB criteria [20,28]. The majority of the aphasic patients were classified as Anomic (*n* = 18), Broca’s aphasia (*n* = 2), Transcortical Motor aphasia (*n* = 2), followed by the remaining other aphasia subtypes (see Table 2). Stroke participants demonstrated adequate auditory comprehension based on the WAB (AC > 60%; see Table 1 for means). Naming performance on the WAB are also provided in Table 2 for each individual stroke patient by lesion subgroups. Three stroke patients with significant auditory comprehension disturbance (< 60 auditory comprehension) and associated severe naming deficits were eliminated as outliers. Control participants were required to have normal language and cognitive performance and adequate performance on experimental tasks for inclusion into the analysis.

### 2.3. Experimental Conditions

The participants were administered an experimental object naming battery developed and described in a study investigating naming performance of younger and older adults [3]. The same common man-made objects with manipulable properties (e.g., toothbrush, saw, zipper, and telephone) were utilized in this study, and contained identical normative properties of verbal fluency, name agreement, alternate response ranges, and familiarity. The properties of the naming battery can be obtained from the original publication and include verbal frequency, familiarity, written frequency and other details. The naming battery presented 25 objects across the following conditions in a counterbalanced order across subjects: (1) Auditory (*A*)—digitized sounds of objects normalized for loudness equivalence; (2) Visual (*V*)—static color photographs of objects without images of motion; and (3) Audiovisual (*AV*)—sounds and photographs of the same objects presented simultaneously. Auditory naming was retested following a brief delay to determine improvement following the experiment in comparison to the first auditory presentation. Auditory2 naming was completed in all LHD patients and a subset of the control group (*n* = 67) 20–30 minutes following Auditory 1 consistent with delayed recall in standardized tests of memory in neuropsychological assessment and our previous paper [3].

Presentation stimulus delivery software program (Neurobehavioral systems, version 10.1) via a Dell laptop and headphones provided 4 second stimulus presentation. Subject responses preceding 4 second stimulus presentation completion were recorded for reaction time by the experimenter. Subjects were allowed a total of 25 seconds to produce a naming response even though the stimulus presentation was only 4 seconds. General or nonspecific responses (e.g., naming a general semantic class of object) were followed-up by the examiner, and self-corrected responses were marked correct when provided in the 25 second time frame. Reaction times were measured from stimulus presentation to first response generation and recorded by the experimenter utilizing the Presentation program developed for the experiment. Response accuracy and reaction times in milliseconds were collected by the experimenter on the laptop. 

### 2.4. Analysis

A multivariate mixed design was conducted with age and education as covariates allowing for inclusion of a within-subjects factor for sensory condition (auditory, visual, and auditory–visual), a between-subjects factor (control versus stroke groups), and the interaction between the two in one model. This model is conservative and controls for multiple comparisons with several dependent measures (accuracy and reaction time) and is not dependent upon sphericity. Univariate values were examined when the multivariate model was significant, followed by post-hoc analyses with Bonferroni correction when univariates were significant. Additional multiple regression analyses were conducted to explore the impact of specific lesion location, aphasia severity, stroke interval, and cognitive dysfunction. 

Between-group factors allowed for the following group comparisons: (i) LHD stroke to normal controls, (ii) LHD lesion locations (CO = Cortical, CO–SC = Cortical–Subcortical, and SC = Subcortical) relative to normal controls. Finally, several stepwise regression models evaluated cognitive, language and lesion variables as predictors of naming performance by condition. 

## 3. Results

### 3.1. LHD versus Controls

A multivariate model compared naming accuracy and reaction times in LHD stroke patients (*n* = 42) relative to controls (*n* = 118) across conditions. A multivariate model utilized age and education as covariates, group (controls, LHD) as the between-subjects factor and sensory condition (auditory, visual, and multisensory) as the within-subjects or repeated factor. The multivariate model was significant for age, *F*(2,155) = 20.6, *p* < 0.0001, but not education, *F*(2,155) = 0.04, *p* < 0.96. The multivariate model revealed significant between-subjects effects for group, *F*(2,155) = 18.5, *p* < 0.0001, within-subjects effects revealing changes in performance across sensory conditions, *F*(4,153) = 2.5, *p =* 0.042, and a sensory by group interaction, *F*(4,153) = 5.3, *p* < 0.0001. 

#### 3.1.1. Group

Univariate tests revealed a significant between-subjects effect of group for naming accuracy, *F*(1,156) = 33.4, *p* < 0.0001, and correct reaction time, *F*(1,156) = 19.6, *p* < 0.0001. Controls had greater naming accuracy (mean difference = 10.9) and faster reaction times for correctly named items (mean difference = 0.77 s) across all conditions relative to LHD patients *p <* 0.0001 (Table 3).

#### 3.1.2. Sensory Condition

Univariate tests displayed significant within-subjects effects for sensory condition on naming accuracy, *F*(2,312) = 3.3, *p* = 0.038, but not for correct reaction times, *F*(2,312) = 1.05, *p* = 0.351. The *A* condition displayed lower accuracy than the *V* (mean difference = −24.2) and *AV* conditions (mean difference = −25.2), *p* < 0.0001. However, the *V* and *AV* conditions did not differ overall in terms of accuracy (mean difference = −0.974, *p* = 0.206; Table 3).

#### 3.1.3. Sensory Condition by Group Interaction

There was also a sensory condition by group interaction for naming accuracy, *F*(2,312) = 14.1, *p* < 0.0001, and for correct reaction times, *F*(2,312) = 3.2, *p* = 0.043.

*Naming Accuracy*. Pairwise comparisons with Bonferroni correction revealed that LHD patients had lower accuracy for each of the sensory conditions relative to controls (mean difference *A* = −18.6, mean difference *V* = −6.8, and mean difference *AV* = −7.5), *p* < 0.001. However, LHD patients had greater differences between auditory naming accuracy relative to visual and multisensory accuracy (mean differences presented in Table 3 and Table 4). Also, while normal controls had significantly higher naming accuracy in the multisensory compared to the visual condition, *p* = 0.045, LHD patients did not differ between the visual and multisensory conditions, *p* = 1.0 (see Table 3 and Table 4). 

*Reaction Times for Correctly Named Items*. Pairwise comparisons with Bonferroni correction revealed that LHD patients had slower reaction times for each of the sensory conditions relative to controls (mean difference *A* = 1.06, mean difference *V* = 0.469, and mean difference *AV* = 0.786), *p* < 0.001. However, LHD patients’ reaction times improved more significantly than controls when visual cues were provided in place of auditory cues, or with the addition to auditory cues in a multisensory condition (see Table 4 for mean differences). 

### 3.2. Lesion Group

A multivariate model compared naming accuracy and reaction times in LHD stroke patients with CO lesions (*n* = 17), CO–SC lesions (*n* = 10), and SC lesions (*n* = 15) relative to controls (*n* = 118) across conditions. The multivariate model was significant for age, *F*(2,153) = 19.9, *p* < 0.0001, but not education, *F*(2,153) = 0.007, *p* < 0.993. The multivariate model revealed significant between-subjects effects for lesion group, *F*(6, 308) = 8.5, *p* < 0.0001, within-subjects effects of sensory conditions, *F*(4,151) = 2.65, *p* = 0.035 and a sensory by lesion group interaction, *F*(12,459) = 2.95, *p* = 0.001. 

#### 3.2.1. Group 

Univariate tests revealed significant between-subjects effects for lesion group on naming accuracy, *F*(3,154) = 16.1, *p* < 0.0001, and correct reaction time, *F*(3,154) = 11.4, *p* < 0.0001.

**Figure 1:**
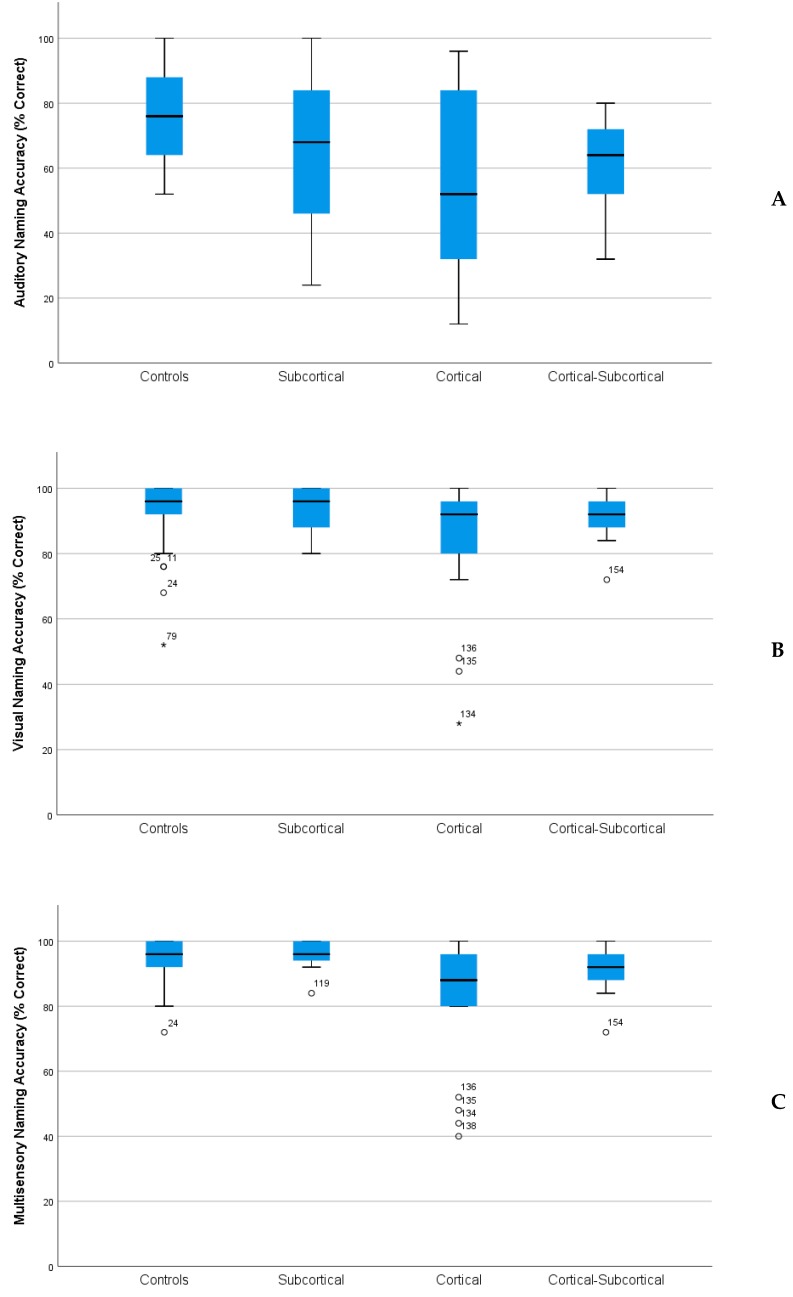
Naming accuracy (% Correct) by group and condition. Box plots depicting median and quartiles, and subject numbers for values extending outside range. **A**: Auditory naming accuracy by LHD Lesion Group. **B**: Visual naming accuracy by LHD Lesion Group. **C**: Multisensory naming accuracy by LHD Lesion Group.

*Naming Accuracy.* Controls had higher naming accuracy than patients with CO (mean difference = 16.1, *p* < 0.0001) and CO–SC lesion locations (mean difference = 10.6, *p* = 0.004), but were not significantly different than SC patients (mean difference = 5.1, *p* = 0.347). SC patients had higher naming than CO patients (mean difference = 11.1, *p =* 0.003), but were not significantly different from CO–SC patients (Figure 1A–C). 

*Reaction Times for Correctly Named Items.* Controls produced faster reaction times than CO and CO-CS patients (CO mean difference = −1.2, *p* < 0.0001; CO–SC mean difference = −0.984, *p* < 0.005), but were not significantly different from the SC group (mean difference = −0.15, *p* = 1.0). The SC group produced faster times than the CO group (mean difference = −1.01, *p* < 0.005), but not the CO-CS group (mean difference = 0.83, *p* < 0.08). 

#### 3.2.2. Sensory Condition

Univariate within-subjects effects were not significant for sensory condition on naming accuracy, *F*(2,308) = 2.7, *p* = 0.07 or for correct reaction time, *F*(2,308) = 1.4, *p* = 0.26 (Table 5). 

#### 3.3.3. Sensory Condition by Lesion Group Interaction

There was a significant within-subjects effects for sensory condition by lesion location interaction for naming accuracy, *F*(6,308) = 5.5, *p* < 0.0001, and correct reaction time, *F*(6, 308) = 1.4, *p* = 0.041. 

*Naming Accuracy.* Controls displayed higher naming accuracy compared with CO group across all conditions, although the differences were largest in the auditory condition. Controls were only significantly different from patients with subcortical involvement on the auditory condition. SC patients had higher naming accuracy for auditory and multisensory conditions relative to the CO patients. None of the lesion groups had significantly different performances in the auditory condition. See Table 5 for mean differences across groups. 

*Reaction Times for Correctly Named Items.* Controls had faster reaction times for correctly named items across conditions relative to patients with cortical involvement (with the exception of visual naming for CO–SC patients). Reaction times were not significantly different between controls and SC patients for any of the conditions. However, the SC group displayed faster reaction times than the CO group for both *V* and *AV* conditions, but they were not faster in auditory naming. The CO-CS group did not differ from the other lesion groups across conditions (Table 5 for significance and mean differences). 

### 3.3. Stepwise Multiple Regression

To investigate the effect of stroke associated variables (stroke interval, language, and cognitive dysfunction) on naming accuracy, three separate regression analyses were conducted for each of the sensory conditions. Predictor variables (age, education, stroke interval, MMSE, WAB-AQ, verbal fluency, and LNS—Letter Number Sequencing) were examined in stepwise regression models predicting auditory, visual, and multisensory naming accuracy. Age accounted for a significant amount of variance in auditory naming accuracy (43.5% of the variance, *p* < 0.001), with the addition of semantic fluency (8.9% variance), LNS (3.7% variance), and MMSE (7.6%) accounting for additional variance (63.7% total variance explained; Table 6). The MMSE explained 74.3% of the variance in visual naming accuracy (*p* < 0.0001), with the addition of LNS accounting for an additional 1.9% variance (76.2% total variance explained). Multisensory naming accuracy was explained by aphasia severity (WAB-AQ, *p* < 0.001), with the MMSE accounting for additional variance (3.9%; 71.1% total variance explained). See Model 1 Table 6. 

**Figure 2:**
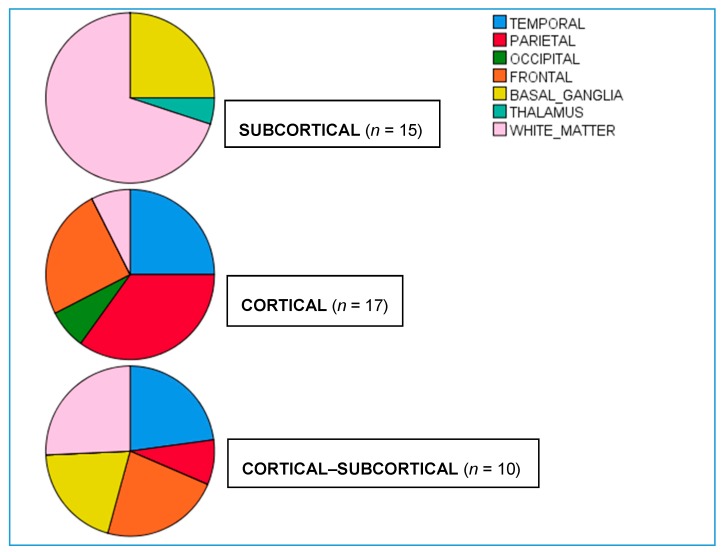
Percent distribution of lesion location for LHD subgroups. Based on the total LHD subgroup (Cortical, Cortical−Subcortical, and Subcortical) with lesion involvement to specific anatomical regions.

In a second set of stepwise regression analyses, the impact of lesion location (temporal, parietal, occipital, frontal lobes, basal ganglia, thalamic, and white matter involvement, Figure 2) were evaluated on naming accuracy across conditions, while controlling for age and education. In Model 2, age accounted for significant variance in both auditory and visual naming accuracy (44.7% and 34.7% variance, respectively), followed by additional variance accounted for by temporal lobe involvement (7.7% and 15.6% variance, respectively). Conversely, multisensory naming accuracy was best predicted by occipital lobe involvement explaining 27% of the variance, with additional variance explained by age (14.5%) and temporal lobe involvement (16.1%; 57.6% total variance explained). See model 2 Table 6. 

#### Control Analyses

Control analyses were conducted on a repeated auditory condition to assess learning, improvement in auditory naming and recognition (i.e., Auditory 2), to assess for impact of peripheral hearing impairment and randomization order on the test results. 

### 3.4. Auditory2 Naming

All LHD patients and a subset of the control subjects (*n* = 68) repeated the *A* naming condition following a 20 to 30 min delay at the end of the experiment. The multivariate mixed model with age and education as covariates, and naming accuracy and correct reaction times as dependent variables evaluated changes in *A* accuracy across times 1 and 2. Age was the only covariate to significantly adjust the variance between groups, *F*(2,104) = 22.79, *p* < 0.0001. There was a significant between-subjects effect for group, *F*(2,104) = 10.75, *p* < 0.0001, but the within-subjects effects for change in naming from initial to delay, *F*(2,104) = 1.97, *p* = 0.15, and condition by group interaction, *F*(2, 104) = 2.46, *p* = 0.09, were not significant. Univariate tests revealed a significant between-subjects effect of group for *A* naming accuracy *F*(1,105) = 21.17, *p* < 0.001, and correct reaction time, *F*(1,105) = 9.27, *p* < 0.005. The results are consistent with lower *A* accuracy for LHD subjects (mean = 69.19) relative to normal controls (82.99) and longer reaction times (LHD = 4.58; controls = 3.64 s) across both initial and delayed conditions. 

### 3.5. Audiometric Analyses

Differences between the left and right ears were computed for each subject at each frequency levels of 250, 500, 1000, 2000, and 4000 Hz. Group comparisons did not reveal differences between controls and LHD patients, *F*(5, 150) = 1.8, *p* = ns. Group comparisons based on stroke location were not significant at any of the frequency levels and thus were not utilized in adjustment of the variance in auditory naming performance. 

### 3.6. Accuracy Analyses for the First Block of Stimulus Items

To evaluate the influence of counterbalancing, we analyzed the first block of each condition as a control analysis. *A* naming performance was higher when the first block was *V* (76.6%) or *AV* (80.3%) compared to *A* first block presentation (60.7%). *V* accuracy was higher when the first block was *A* (94.8), or *AV* (94.5%) compared to *V* (89.7%). The first block condition did not significantly impact *AV* accuracy. However, LHD CO patients had lower *A* naming accuracy irrespective of the first block order.

## 4. Discussion

This study evaluated lexical retrieval performance across auditory (nonverbal) and visual modalities in older healthy adults relative to left hemisphere stroke patients. We predicted there would be significant variability across naming conditions related to degree of language deficits following stroke and based on lesion location. Variability across patients clarify neural mechanisms mediating modality specificity as well as multisensory integration in lexical retrieval. Investigation of how language deficits and lesion location influence lexical retrieval under different sensory conditions provides insights into post-stroke intervention potential. Because of variability in deficits in cognition and language and lesion location, future therapeutic interventions should be tailored to patient characteristics to obtain optimal treatment outcomes [29]. 

Healthy adults produced more accurate and more rapid responses in naming compared to patients with left hemisphere stroke. There was also a sensory condition effect, with all subjects producing less accurate and slower naming responses in the auditory condition compared to the visual and multisensory conditions. Across groups, there was substantial performance improvement in accuracy when naming to visual cues compared to auditory cues. Past studies have typically relied on visual confrontation naming to assess persisting naming deficits. Consistent with these previous results, our study revealed that patients with cortical lesions were more impaired than those with subcortical lesions on both visual and multisensory naming, although they did not differ from patients with cortical−subcortical lesions. Conversely, auditory naming was the most sensitive to detecting deficits and revealed lower performance than other conditions for all subjects. Cortical and cortical−subcortical patients performed worse than healthy controls on auditory naming, suggesting auditory naming may provide increased sensitivity in detecting persisting lexical retrieval deficits across lesion groups. Although patients with cortical lesions had lower naming accuracy on visual and multisensory naming, they did not have significantly lower performance on auditory naming. 

### 4.1. Lesion Location and Lexical Retrieval

Cerebrovascular insults often affect the middle cerebral artery constraining the distribution of affected perisylvian regions impacting language dysfunction. Comparison to other types of stroke such as those limited to subcortical regions can help elucidate critical regions involved in lexical retrieval. Left hemisphere stroke is frequently associated with language impairments, and there is evidence that damage to the mid-posterior inferior temporal cortex and underlying connections result in lexical retrieval deficits [15]. Extent of disconnection from language regions in the inferior frontal region has demonstrated predictive validity for degree of naming deficits and may account for persisting naming deficits following subcortical stroke [30]. Similar to results of lexical retrieval following neurosurgical removal of glioma, our findings support a role for the subcortical network in mediating lexical retrieval although the contribution is less robust and may depend on the degree of underlying white matter disconnection [15]. 

Our findings are similar to previous studies and support the critical role of the temporal lobe in naming irrespective of sensory modality, and suggest that visual confrontation naming may rely on different neural mechanisms than either auditory naming or audiovisual integration. The finding of significantly lower scores on visual confrontation naming exclusively in patients with cortical lesions, argues that visual naming is mediated by a less distributed network. Thus, patients with left hemisphere stroke with milder lexical-retrieval declines may go undetected on typical tests of visual confrontation naming. Conversely, auditory confrontation naming may be more sensitive to milder deficits and capable of detecting naming deficits in other lesion locations. Although cognitive functioning and temporal lobe involvement predicted naming accuracy across conditions, auditory cues required additional processing demands. Auditory naming was more impacted by age effects and semantic capacity compared with the other conditions. Our findings support left cortical localization of object semantics unrelated to discrimination of acoustically related sounds [31]. Future investigations are needed to delineate the distributed network for naming in healthy individuals, although our findings confirm the critical role of the temporal lobe in lexical retrieval irrespective of modality of elicitation. 

Evidence from fMRI and lesion symptom mapping in aphasia suggest that verbal and nonverbal auditory processing relies on overlapping cortical regions within the left hemisphere, whereas there is greater activation in the right hemisphere for processing environmental sounds [32,33]. However, the distribution of auditory verbal and auditory nonverbal (sound) neural networks has not been well characterized. Studies of patients with lateralized lesions have displayed dissociation between auditory recognition and localization, and motion perception suggesting a lateral recognition pathway and a medial and posterior spatial pathway [34]. Furthermore, the association between nonverbal auditory recognition and linguistic processing has not been clearly established, although it has been considered that auditory recognition proceeds in parallel as opposed to hierarchical processing [35]. 

A distributed frontal, parietal, and temporal activation network has been identified for unimodal auditory and visual stimuli, while posterior superior temporal sulcus/middle temporal gyrus, dorsal lateral prefrontal cortex, and ventral temporal cortex in previous studies associated to audiovisual stimuli [17]. In our study, integration of simultaneous congruent information from a second modality (auditory added to visual) relied on intact left cortical functioning since efficient multisensory lexical retrieval was only apparent for healthy older adults. Furthermore, multisensory integration in LHD patients was predicted by severity of language impairment, and lesion involvement of the temporal and occipital lobes. Language processing can be conceptualized in two different systems (sensory−conceptual and sensory−motor) that rely on auditory cortices interacting with projections to the temporal lobe and with the motor system to temporal parietal regions (dorsal stream) [36,37]. Similarly, our findings support the premise that the visual system interfaces with conceptual representations of objects through ventral (occipitotemporal) and dorsal object recognition streams (occipitoparietal) [38]. Our results of predictors of multisensory performance in LHD stroke support findings from neuropsychological lesion studies and fMRI investigations providing evidence that the processing of visual object categories is mediated by an occipitotemporal network [17,39,40,41]. 

There are numerous investigations focused on audiovisual integration in speech processing that have revealed critical contributions from the auditory cortex, the posterior superior temporal sulcus (STS), and the posterior inferior frontal gyrus [16,17,18]. In addition to these regions, lesion studies have also implicated lateral occipital visual cortex in addition to multisensory regions in the STS as critical to multisensory speech integration [4]. Unlike multisensory speech integration, the overlapping network mediating auditory verbal and nonverbal (sound) naming has not been well characterized. Thus, the brain regions impacting audiovisual integration in lexical retrieval warrant further elucidation to better characterize lexical retrieval and develop potential intervention models.

### 4.2. Reaction Times in Naming

Simultaneous processing of audiovisual cues increases reaction times, even when accounting for accuracy. This is consistent with increased cognitive demands required for simultaneous processing of audiovisual information, and variance in multisensory naming was predicted by general cognitive capacity as well as degree of language severity. These findings are not supportive of integration across modalities, but demonstrate an increased cognitive load required for dual processing of sensory data. This suggests that the left cortex is critically involved in multisensory processing required for improved efficiency, and is reflected in the more significant increase in reaction time for LHD patients. There were longer reaction times for patients with cortical stroke across conditions relative to controls and subcortical stroke patients, even when accounting for reaction times for correct items. Although cortical patients had longer reaction times than subcortical patients in the auditory condition, this did not reach statistical significance likely related to the small sample sizes and variability in performance. The cortical patients displayed increased reaction times across experimental conditions related to increased information processing demands for accessing semantics unrelated to auditory processing demands. Therefore, additional information from a second modality was not integrated effectively and there were no gains or multisensory enhancement in lexical retrieval following left cortical damage. The reaction times were not significantly different between any of the other groups irrespective of experimental condition. 

### 4.3. Lesion Size & Characteristics

Differences in lesion size contribute to the severity of cognitive and language deficits across lesion groups since cortical strokes are larger than subcortical strokes. Previous investigations focused on post-stroke aphasia have documented clear impact of lesion size, although many of these investigations have relied on gross estimates of size (e.g., small, medium, and large) as opposed to precise volumetric measurements [30,42]. The full extent of brain regions affected following stroke is likely underappreciated by visual inspection alone, and is influenced by other factors including disconnection and hemodynamic changes [43,44]. Therefore, covarying for lesion size may be insufficient to fully account for functional differences in language, especially since size and location are correlated and could obscure the impact of lesion location on pattern of deficits. 

Comparison of cortical lesions to cortical−subcortical and subcortical lesions provides some estimation of how lesion size and location impact severity and naming profiles. Our study findings revealed that both larger lesions (cortical−subcortical) as well as smaller lesions with subcortical involvement had greater accuracy in visual confrontation naming compared to patients with lesions confined to the cortex (larger lesions). These findings argue against lesion size alone as being able to account for naming accuracy, and the most severe deficits resulted from disruption of critical language regions in the left cortex. We also recognize that the stroke testing interval impacts degree of recovery and severity of both cognitive and language deficits. While stroke interval was not predictive in any of regression models, degree of language and cognitive deficits were predictive and may reflect some of the overlapping variance in this predictor variables. Similarly, longer stroke-testing intervals have less influence on the severity of deficits in larger lesions than smaller lesions, recognizing the interplay between lesion size, chronicity, and persisting deficits. Nonetheless, we acknowledge that lesion size contributes to the severity of language deficits, and thus one study limitation is the absence of detailed volumetric and morphometric measurements. Therefore, our study findings should be substantiated with future investigations carefully controlling for both detailed anatomical distribution of lesions, lesion size as well as functional imaging measurements. 

### 4.4. Aging & Multisensory Processing

Age-associated auditory processing difficulties are common, although complex auditory processing deficits are distinct from hearing insensitivity. This is supported by equivocal hearing sensitivity across frequencies between left hemisphere stroke patients and healthy controls, and the amount of variance age accounted for in auditory accuracy. However, age accounted for significant variance in naming accuracy in most of our predictive models and not just for auditory naming. Therefore, hearing sensitivity is unable to account for differences in auditory naming between groups, and emphasizes the higher level of auditory processing required for successful lexical retrieval. 

The ability to integrate in a multisensory fashion may be a critical marker of successful aging [45]. This can be related to variable aging including extent of brain atrophy, or degree of plasticity that can facilitate integration of audiovisual information that can access semantics and/or lexical representations [45,46]. Age-related changes in morphology implicate a critical role of the left superior temporal cortex volume in predicting semantic performance over time in healthy older adults [47]. There is some indication both temporal acuity and multisensory integration decline with aging [48]. This is supported by age-related changes in width and depth of cortical sulci demonstrating more profound age-related changes in multisensory as opposed to unisensory cortical regions [49]. Contrary to this evidence for declines in temporal acuity in aging, our current findings and previous work demonstrate improvements with multisensory naming evident only in older adults [3]. In summary, integration of multiple sensory environmental signals (audiovisual) into one percept relies heavily on the left superior temporal region which is susceptible to increased atrophy in aging or neurodegeneration as well as the occipital region for processing visual information [47,49,50].

## 5. Conclusions

Processing of environmental sounds may share overlapping neural networks with language related to the semantic categorization and skilled movements in the left hemisphere that also have a strong temporal component. There is evidence that correctly categorizing tool sounds activates a distributed left hemisphere cortical network that overlaps with tool manipulations that require integration of spatial and temporal features, suggesting that sound recognition may involve not only a “what” pathway, but also a “how” pathway associated with the motor actions that produce the sound [19,51]. It remains unclear whether this auditory network partially overlaps with visual processing networks related to biological motion and visual recognition of tools associated with action, which might presume multisensory processing of object semantics [19]. Although our results are capable of confirming a critical role of the left cortex in multisensory integration, stroke survivors with left cortical involvement and aphasia are less likely to benefit from multisensory cues in terms of naming efficiency or accuracy. 

Multisensory cues that result in behavioral advantages require overlap of both spatial and temporal information that optimize integration across the senses. Study results indicate that outcomes can improve with multisensory information with respect to detection, localization as well as speed of responding if critical regions in the left cortex are spared [52,53,54]. Our results support involvement of a distributed left hemisphere network for auditory naming that overlaps with a tool-use distributed cortical−subcortical network mediating spatiotemporal features of action associated with environmental sounds (how pathway) [51,55]. 

## Figures and Tables

**Table 1:** Characteristics of left hemisphere stroke patients.

#	Age	Sex	Stroke Interval	WAB AQ	Aphasia Type	WAB Naming	WAB Aud. Comp.	Lesion Location
**CO**								
**1**	75	Male	32.95	94.4		9.5	100	Frontal and parietal lobes and deep white matter
**2**	77	Male	20.14	78.7	Anomic	7.4	80	Frontal, temporal, and parietal lobes
**3**	74	Male	16.03	86.9	Anomic	9.8	99	Middle and posterior frontal gyri, precentral, postcentral, and posterior parietal gyrus
**4**	50	Male	20.3	76.1	Transcor. Motor	8.3	96	Superior temporal gyrus extending to the perirolandic region
**5**	50	Male	19.87	94.9		9.8	99	Anterior parietal lobe, extending anteriorly and inferiorly including Wernicke’s area
**6**	67	Female	5.81	80.8	Anomic	9.0	91	Temporal lobe
**7**	40	Male	59.53	73.5	Conduction	8.7	83	Middle cerebral artery territory
**8**	50	Female	9.49	81.2	Anomic	8.3	87	Insula, operculum, and punctate foci within the frontal and parietal lobes
**9**	77	Female	7.29	75.1	Transcor. Motor	7.4	93	Posterior parietal and superior temporal lobes
**10**	78	Male	27.66	36.8	Broca’s	4.0	67	Posterior parietal lobe extending into the medial temporal lobe/subinsular cortex
**11**	78	Male	28.41	91.4	Anomic	9.4	97	Insular cortex, superior frontal, and lateral parietal lobes
**12**	47	Male	14.09	84.4	Anomic	8.1	82	Posterior parietal and occipital lobes
**13**	52	Male	41.39	95.6		9.8	100	Posterior frontal lobe
**14**	85	Male	41.39	58.5	Wernicke’s	5.0	65	Occipital and posterolateral aspect of temproparietal lobes
**15**	62	Male	18.39	97.0		9.2	94	Posterior territory of the left middle cerebral artery; lacunar infarct
**16**	56	Female	17.47	46.0	Broca’s	4.6	81	Temporal and parietal occipital lobes
**17**	44	Male	4.13	96.1		10		Distal middle cerebral and left anterior cerebral artery; lateral ventricle in posterior horn
**CO–SC**								
**18**	52	Female	14.58	92.1	Anomic	8.7	98	Insula, subinsular white matter, perisylvian frontal & temporal lobe, and body of caudate
**19**	56	Male	38.01	85.0	Anomic	8.0	89	Frontal and anterior superior temporal lobes, insula, and corona radiata
**20**	38	Female	11.17	97.2		9.8	100	Caudate head, internal capsule, putamen, perisylvian cortex, deep white matter, and parenchyma adjacent to the atria of lateral ventricle
**21**	57	Female	21.12	87.8	Anomic	9.5	96	Posterior putamen extending to the corona radiata. Small focus of restricted diffusion noted in the periatrial region of temporal lobe
**22**	52	Male	20.07	84.5	Anomic	8.7	96	Multiple small foci of restricted diffusion within MCA, ACA, and deep watershed territories. Acute basal ganglia infarct.
**23**	44	Male	44.12	94.3		9.7	94	Caudate head, basal ganglia, posterior frontal, parietal, and temporal lobes
**24**	55	Female	7.03	95.5		9.6	96	Frontal lobe and corona radiata
**25**	74	Female	7.32	78.2	Anomic	8.3	92	Frontal lobe, and subinsular region, putamen, and external capsule
**26**	64	Female	14.48	91.2	Anomic	8.9	97	Sylvian and perisylvian cortex, precentral and postcentral gyrus, posterior aspects of superior and middle frontal gyrus, caudate head and body, putamen, and external capsule
**27**	69	Male	28.05	83.1	Anomic	9.3	98	Subinsular region, posterior temporal lobe, centrum semiovale, corona radiata, and parietal lobe
**SC**								
**28**	60	Male	38.34	93.2	Anomic	9.6	100	Basal ganglia extending into corona radiata
**29**	65	Male	25.49	95.7		8.7	99	Posterior limb of the internal capsule, inferior putamen, and anterior periatrial white matter
**30**	64	Female	26.25	96.0		9.2	100	Posterior limb of the internal capsule
**31**	65	Male	145.81	85.2	Anomic	9.6	98	Corona radiata
**32**	51	Male	7.85	85.5	Anomic	9.2	96	Caudate body, corona radiata, and putamen
**33**	64	Male	12.71	93.8		9.6	93	Paramedian pons
**34**	63	Male	26.94	83.9	Anomic	8.9	96	Posterior limb of the internal capsule
**35**	48	Female	31.27	95.4		9.8	100	Thalamic lacunar infarct
**36**	70	Male	12.0	99.0		9.5	100	Anterior limb of internal capsule
**37**	70	Male	4.59	81.4	Anomic	7.6	91	Posterior limb of the internal capsule
**38**	64	Female	39.55	98.3		9.7	99	Basal ganglia and centrum semiovale
**39**	62	Male	33.8	97.2		9.8	94	Pons
**40**	63	Male	1.6	98.8		9.8	100	Medullary infarct
**41**	47	Female	61.63	99.6		9.8	100	Posterior putamen
**42**	58	Female	19.54	97.1		9.8	96	Pontomedullary junction

Note. CO = Cortical Stroke location; CO–SC = Cortical–subcortical stroke location; SC = Subcortical stroke location; Stroke Interval = Interval in months from stroke to evaluation; WAB Quotient = Western Aphasia Battery Aphasia Quotient in percentage points; WAB AQ < 93.7 presence of aphasia; WAB Aud. Comp. = Western Aphasia Battery Auditory Comprehension in percentage points [20]; Aphasia = Aphasia Subtypes from the Western Aphasia Battery; WAB Naming = Score out of possible 10; MMSE = Mini-Mental State Examination; BNT = Boston Naming Test; FAS = Controlled Oral Word Association.

**Table 2:** Subject characteristics and screening measures (means).

	HC (*n* = 118)	LHD (*n* = 42)	*F*	Sig. *p*
**Age**	63.1 (9.5)	60.4 (11.5)	2.3	0.133
**Education**	16.5 (2.5)	13.4 (2.4)	51.3	0.0001
**MMSE**	29.2 (0.9)	25.5 (4.8)	66.4	0.0001
**WAB AQ**	98.4 (2.3)	86.6 (13.5)	83.9	0.0001
**Boston Naming**	51.8 (11.9))	40.5 (14.1)	25.7	0.0001
**Phonemic Fluency**	49.5 (9.5)	33.5 (13.3)	70.7	0.0001
**Semantic Fluency**	52.1 (9.5)	40.7 (13.3)	35.5	0.0001

Note. HC = Healthy Controls; LHD = Left Hemisphere Damage; Means (SDs); MMSE = Mini-Mental State Examination; WAB AQ = Western Aphasia Battery Aphasia Quotient (out of 100); Boston Naming = Boston Naming Test (T score); Phonemic (FAS); Semantic (animals) Fluency (T scores); *F* = F statistical value; Sig. *p* = Significance as a probability.

**Table 3:** Mean (SDs) of naming accuracy (%) and reaction time (seconds) by condition.

		Accuracy			Reaction	Time
	*Auditory*	*Visual*	*Multisensory*	*Auditory*	*Visual*	*Multisensory*
**Controls**	75.2	94.6	96	3.93	2.03	2.40
(*n* = 118)	(13.1)	(7.3)	(5.1)	(1.4)	(0.52)	(0.72)
**LHD**	60.7	87.9	88.4	4.86	2.57	3.14
(*n* = 42)	(24.5)	(15.5)	(15.5)	(1.8)	(0.73)	(1.1)
**Cortical**	56.5	81.7	80.5	5.19	3.04	3.56
(*n* = 17)	(29.6)	(21.5)	(20.9)	(1.9)	(0.84)	(1.5)
**Cortical–Subcortical**	62.7	90.5	90	5.52	2.49	3.17
(*n* = 10)	(14.8)	(8.3)	(7.8)	(1.8)	(0.41)	(0.91)
**Subcortical**	64.3	93.3	96.3	4.03	2.07	2.65
(*n* = 15)	(23.9)	(6.9)	(4.7)	(1.3)	(0.33)	(0.57)

Naming accuracy based on percent correct. Reaction time in seconds for correctly named items.

**Table 4:** Mean differences for naming accuracy and correct reaction time across conditions.

	*V* vs. *A*	*MS* vs. *A*	*MS* vs. *V*
**ACCURACY**			
*Controls*	18.3 ***	19.7 ***	1.3 *
*LHD*	30.1 ***	30.7 ***	0.60
**REACTION TIME**			
*Controls*	−1.8 ***	−1.5 ***	0.32 ***
*LHD*	−2.4 ***	−1.8 ***	0.63 ***

Based on estimated marginal means with age and education as covariates. Adjustment for multiple comparisons Bonferroni. Note: Bold values with * significance *p* < 0.05; ** significance *p* < 0.01; *** significance *p* < 0.001 denoting significance between groups. *A* = Condition; *V* = Visual; *MS* = Multisensory. Naming accuracy based on percent correct. Reaction time in seconds for correctly named items.

**Table 5:** Mean group differences for naming accuracy and correct reaction time across conditions.

	HC vs. CO	HC vs. CO–SC	HC vs. SC	SC vs. CO	SC vs. CO–SC	CO vs. CO–SC
**ACCURACY**						
*Auditory*	21.2 ***	19.5 **	14.8 **	6.5	4.7	−1.8
*Visual*	12.3 ***	5.6	1.0	11.3 **	4.6	−6.7
*Multisensory*	14.8 ***	6.8	−0.73	15.5 ***	7.6	−7.9
**REACTION TIME**						
*Auditory*	−1.4 **	−1.7 **	−0.22	−1.15	−1.51	−0.36
*Visual*	−0.93 ***	−0.41	−0.04	−0.97 ***	−0.454	0.52
*Multisensory*	−1.2 ***	−0.81 *	−0.28	−0.91 **	−0.532	0.38

Based on estimated marginal means with age and education as covariates. Adjustment for multiple comparisons Bonferroni. Note: * significance *p* < 0.05; ** significance *p* < 0.01; *** significance *p* < 0.001 denoting significance between groups in bold. SC = Subcortical; CO–SC = Cortical–Subcortical; CO = Cortical. Naming accuracy based on percent correct. Reaction time in seconds for correctly named items.

**Table 6:** Stepwise multiple regression results of predictors of naming accuracy.

Dependent Variable	Predictor	Beta	SE	Stand. Beta	*t*	*p*	R^2^	Adj. R^2^
**MODEL 1**								
Auditory								
Accuracy	*1 Age*	−0.925	0.245	−0.431	−3.78	0.001	1 0.450	1 0.435
	*2 Semantic Fluency*	1.13	0.461	0.309	2.46	0.019	2 0.548	2 0.524
	*3 WAIS−IV LNS*	−4.29	1.29	−0.407	−3.31	0.002	3 0.595	3 0.561
	*4 MMSE*	2.15	0.734	0.431	2.93	0.006	4 0.674	4 0.637
Visual								
Accuracy	*1 MMSE*	3.18	0.315	0.986	10.07	0.0001	1 0.749	1 0.743
	*2 WAIS−IV LNS*	−1.36	0.668	−0.200	−2.04	0.048	2 0.775	2 0.762
Multisensory								
Accuracy	*1 WAB-AQ*	0.619	0.166	0.537	3.73	0.001	1 0.680	1 0.672
	*2 MMSE*	1.15	0.464	0.359	2.49	0.017	2 0.726	2 0.711
**MODEL 2**								
Auditory								
Accuracy	*1 Age*	−1.47	0.233	−0.690	−5.78	0.0001	1 0.461	1 0.447
	*2 Temporal Lobe*	−14.6	5.41	−0.295	−2.69	0.01	2 0.548	2 0.524
Visual								
Accuracy	*1 Age*	−0.640	0.173	−0.474	−3.71	0.001	1 0.211	1 0.191
	*2 Temporal Lobe*	−12.9	4.01	−0.411	−3.22	0.003	2 0.380	2 0.347
Multisensory								
Accuracy	*1 Occipital Lobe*	−27.14	6.19	−0.457	−4.39	0.0001	1 0.288	1 0.270
	*2 Age*	−0.558	0.139	−0.413	−4.01	0.0001	2 0.444	2 0.415
	*3 Temporal Lobe*	−12.81	3.27	−0.408	−3.92	0.0001	3 0.607	3 0.576

Dependent Variables: Auditory Naming Accuracy (% Correct); Visual Naming Accuracy (% Correct); Multisensory Naming Accuracy (% Correct). Model 1 Predictor Variables: Age; Years of Education; Stroke−test Interval in Months; MMSE = Mini-Mental State Examination; WAB−AQ = Western Aphasia Battery Aphasia Quotient; FAS Verbal Fluency; Semantic Fluency (animals); WAIS−IV Letter Number Sequencing. Model 2 Predictor Variables: Age; Years of Education; Temporal Lobe, Parietal Lobe, Occipital Lobe, Frontal Lobe, Basal Ganglia, Thalamus, White Matter Involvement.
